# Domain associated with zinc fingers‐containing NF90‐NF45 complex inhibits m^6^A modification of primary microRNA by suppressing METTL3/14 activity

**DOI:** 10.1002/2211-5463.70173

**Published:** 2025-12-06

**Authors:** Takuma Higuchi, Shunsuke Morioka, Keiko Morisawa, Kazutsugu Matsukawa, Shingo Ashida, Takeshi Suzuki, Shuji Sakamoto

**Affiliations:** ^1^ Laboratory of Molecular Biology, Science Research Center, Kochi Medical School Kochi University Japan; ^2^ Research and Education Faculty, Multidisciplinary Science Cluster, Life and Environmental Medicine Science Unit Kochi University Japan; ^3^ Department of Urology, Kochi Medical School Kochi University Japan; ^4^ Division of Functional Genomics, Cancer Research Institute Kanazawa University Japan

**Keywords:** miRNA biogenesis, RNA binding protein, RNA metabolism, RNA methylation, RNA methyltransferase, RNA modification

## Abstract

*N* ^6^‐methyladenosine (m^6^A) modifications accelerate microRNA (miRNA) biogenesis by promoting the processing of m^6^A‐modified primary miRNAs (pri‐miRNAs). However, the regulatory mechanism of m^6^A modification of pri‐miRNA remains unclear. Here, we found that NF90‐NF45 acts as a negative regulator of the m^6^A modification of pri‐miRNA by methyltransferase‐like 3/14 (METTL3/14). Using overexpression constructs, METTL3/14 promoted the biogenesis of *miR‐7*, whereas NF90‐NF45 suppressed miR‐7 biogenesis. METTL3/14 overexpression relieved the inhibition of *miR‐7* biogenesis by NF90‐NF45. NF90‐NF45 attenuated m^6^A modification of *pri‐mir‐7‐1 in vitro*; however, it had no effect on the m^6^A modification of *pri‐mir‐200a* because of the lower binding affinity of *pri‐mir‐200a* to NF90. Furthermore, NF90‐NF45 did not interact with METTL3/14, according to immunoprecipitation analysis. These findings suggest that the m^6^A modification of pri‐miRNAs by METTL3/14 is regulated by NF90‐NF45 competing for pri‐miRNA binding.

## Graphical abstract

NF90–NF45 functions as a negative regulator of methyltransferase‐like 3/14 (METTL3/14)‐mediated *N* ^6^‐methyladenosine (m^6^A) modification on primary microRNAs (pri‐miRNAs). NF90–NF45 binds to anti‐oncogenic pri‐miRNAs and inhibits their m^6^A modification, thereby suppressing the biogenesis of anti‐oncogenic miRNAs. This study demonstrates that competitive binding between NF90–NF45 and METTL3/14 to anti‐oncogenic pri‐miRNAs regulates m^6^A‐dependent microRNA maturation.

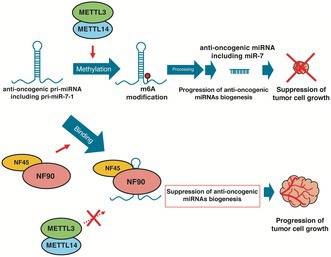

**Abbreviations**
DGCR8: DiGeorge syndrome critical region 8
GAPDH: glyceraldehyde 3‐phosphate dehydrogenase
GST: glutathione *S*‐transferase
HCC: hepatocellular carcinoma
HEK293: human embryonic kidney 293 cells
lncRNA: long non‐coding RNA
m^6^A: *N* ^6^‐methyladenosine
METTL: methyltransferase like
miRNA: micro RNA
ncRNA: non‐coding RNA
PBST: 1× PBS buffer containing 0.1% Tween 20
pre‐miRNA: precursor miRNA
pri‐miRNAs: primary miRNAs
qRT‐PCR: quantitative reverse transcription‐PCR
RNU6B: RNA, U6 small nuclear 6, pseudogene
WTAP: Wilms' tumor 1‐associated protein

*N* ^6^‐methyladenosine (m^6^A) is a common modification of RNA molecules [including mRNA and non‐coding RNA (ncRNA)] in eukaryotes. Over 7000 transcripts in human cells contain m^6^A modifications, according to transcriptome‐wide m^6^A‐sequencing analysis [1]. m^6^A modification regulates mRNA decay/stabilization, splicing, export and long non‐coding RNA (lncRNA) processing [2]. This modification occurs in the consensus DRACH sequence (where D = A, G or U; R = G or A; and H = A, C or U) in RNAs [1, 3]. In mammals, m^6^A modification is controlled by m^6^A writer proteins: methyltransferase‐like 3 (METTL3), METTL14 [4] and Wilms tumor 1‐associated protein (WTAP) [2, 5]. Meanwhile, m^6^A eraser enzymes consisting of the demethylases AlkB homolog 5 [6] and fat mass and obesity‐associated protein [7] reversibly remove these m^6^A modifications. Additionally, m^6^A‐modified RNA is recognized by m^6^A reader proteins, such as the YTH domain‐containing proteins [8] and several heterogeneous nuclear ribonucleoproteins [9, 10]. The writers, erasers and readers control post‐transcriptional gene expression and influence biological events including pathological processes such as cancer [2]. However, the regulatory mechanism underlying m^6^A modification activity of the METTL3/METTL14 complex (METTL3/14) remains unclear.

MicroRNAs (miRNAs) are ncRNAs that repress gene expression by binding to the 3′ untranslated region of the target mRNA. The canonical miRNA biogenesis pathway involves two sequential processes. miRNA genes are initially transcribed by RNA pol II as long primary miRNAs (pri‐miRNAs), which are specific types of lncRNAs. In the nucleus, the microprocessor complex containing Drosha (an RNase III enzyme) and DiGeorge syndrome critical region 8 (DGCR8) (a dsRNA‐binding protein) performs the initial processing events involving the processing of pri‐miRNA into precursor miRNA (pre‐miRNA). Pre‐miRNA is exported from the nucleus to the cytoplasm by exportin‐5 and RanGTP transporter. Subsequently, the pre‐miRNAs are processed to miRNA duplexes by Dicer (another RNase III enzyme) and TRBP (i.e. trans activation response RNA binding protein; an RNA‐binding protein) in the cytoplasm. The duplexes are loaded into the RNA‐induced silencing complex following the release of one strand of the miRNA duplex, leading to the formation of mature miRNAs [11]. m^6^A modification accelerates miRNA biogenesis by promoting the processing of m^6^A‐modified pri‐miRNA [12]. In mammalian cells, pri‐miRNAs are methylated by METTL3. Subsequently, m^6^A reader protein HNRNPA2B1 (i.e. heterogeneous nuclear ribonucleoprotein A2B1) recruits the microprocessor complex Drosha/DGCR8 to the m^6^A‐modified pri‐miRNA to promote miRNA biogenesis [10]. These observations suggest that m^6^A modification of pri‐miRNA initiates miRNA biogenesis. We previously reported that the complex of the proteins nuclear factor 90 (also referred to as ILF3, DRBP76 and NFAR1) and nuclear factor 45 (NF90‐NF45) negatively regulates the pri‐miRNA processing step, resulting in lower mature miRNA production *in vitro* and *in vivo* [13, 14, 15]. Moreover, we reported that NF90‐targeted pri‐miRNAs are highly stable, with lower free energy and fewer mismatches compared to all pri‐miRNAs [16].

m^6^A modifications of pri‐miRNA enhance the processing activity of pri‐miRNA to pre‐miRNA, resulting in the upregulation of miRNA biogenesis [10, 12]. However, the regulatory mechanism of m^6^A modification of pri‐miRNAs remains obscure. In this study, we found that NF90‐NF45 suppressed m^6^A modification of pri‐miRNAs by METTL3/14 *in vitro*. Furthermore, the inhibitory effect of NF90‐NF45 on m^6^A modification depended on a preference of NF90 for pri‐miRNA structures. These findings support the idea that NF90‐NF45 is a competitive inhibitor of METTL3/14 for pri‐miRNA binding that results in impaired m^6^A modification by the enzyme.

## Materials and methods

### Cells and cell culture

HepG2 (RRID:CVCL_0027) cells and human embryonic kidney 293 cells (HEK293) (RRID:CVCL_0045) cells were obtained from ATCC (Manassas, VA, USA) and JCRB (Ibaraki, Osaka, Japan), respectively. The identify of the cell lines were authenticated within the past 3 years by short tandem repeat profiling, performed by BEX Co., Ltd (Itabashi‐ku, Tokyo, Japan). All experiments were carried out using cells confirmed to be free of mycoplasma contamination, tested using MycoStrip (rep‐mys‐10; InvivoGen, San Diego, CA, USA). Dulbecco's modified Eagle's medium‐high glucose (Product No. D5796; Merck, Darmstadt, Germany) supplemented with 10% fetal calf serum was used to culture the HepG2 and HEK293 cells.

### Plasmids

The expression vectors of NF90 (pcDNA3.1‐Flag‐NF90b or pEBMulti‐Neo‐NF90b) and NF45 (pEBMulti‐Hyg‐NF45) were prepared as previously described [13, 15]. To construct expression plasmids of METTL3 and METTL14, cDNAs derived from total RNA extracted from Huh7 cells, which is the human hepatocellular carcinoma (HCC) cell line, were generated and amplified by PCR. These cDNAs were then subcloned into the *Sal*I and *Not*I sites (METTL3) or *Xho*I and *Bam*HI sites (METTL14) of the pEBMulti‐Neo‐TARGET tag vector (Product No. 162‐26531; Wako, Kyoto, Japan).

### Immunoblot analysis

Previous methods were employed for immunoblot analysis [17] and preparation of rabbit polyclonal antibodies: Anti‐NF90 [13] and Anti‐NF45 [15]. Anti‐glyceraldehyde 3‐phosphate dehydrogenase (GAPDH)‐, anti‐FLAG‐tag‐, anti‐METTL3‐ and anti‐METTL14 antibodies were obtained from Wako (Product No. 016‐25523), Merck (Product No. F3165) and Proteintech (Rosemont, IL, USA) (Product No. 15073‐1‐AP and 26158‐1‐AP), respectively. Images were captured and the intensities of specific bands were measured using a FUSION chemiluminescence imaging system (Vilber Bio Imaging, Collégien, France) and Odyssey (LI‐COR Biosciences, Lincoln, NE, USA).

### Quantitative reverse transcription‐PCR (qRT‐PCR)

qRT‐PCR analysis was performed as previously described [15]. Briefly, PCR samples were composed of diluted cDNA (1 : 10), Power SYBR Green Master mix (Thermo Fisher Scientific, Waltham, MA, USA) and 0.5 μm of each of the forward and reverse primers in a total volume of 10 μL. β‐actin was used as the internal control. Transcript levels following qRT‐PCR were quantified using a StepOne Plus real‐time PCR system (Thermo Fisher Scientific). The amplification primers used were: *pri‐mir‐7‐1*, 5′‐GCT GCA TTT TAC AGC ACC AA‐3′ and 5′‐AAA ACT GCT GCC AAA ACC AC‐3′; β‐actin, 5′‐GAG GCC CAG AGC AAG AGA GG‐3′ and 5′‐TAC ATG GCT GGG GTG TTG AA‐3′.

A TaqMan miRNA assay kit and TaqMan gene expression master mix (Thermo Fisher Scientific) were used for qRT‐PCR detection of mature miRNA in accordance with the manufacturer's protocol. A human small nuclear RNA, U6 small nuclear 6, pseudogene (*RNU6B*) was used as an internal control to normalize RNA input.

### Transfection

The transfection of expression plasmids was performed as previously described [15].

HepG2 and HEK293 cells were transfected with NF90, NF45, METTL3, METTL14 and *pri‐mir‐7‐1* expression plasmids using X‐tremeGENE HP DNA transfection reagent (Roche Diagnostics, Basel, Switzerland) in accordance with the manufacturer's instructions.

### Dot blot assay of *in vitro* m^6^A modification

The pri‐miRNA template plasmids for *in vitro* transcription and recombinant proteins [NF90, NF45 and glutathione *S*‐transferase (GST)‐S3a] were prepared as previously described [13, 16, 18].

The *pri‐mir‐7‐1* A175T was amplified from pGEM‐T‐*pri‐mir‐7‐1* [15] by PCR with specific primer set. The first PCR product was used as a template for the second PCR, and the resulting amplified fragment was subsequently cloned into the pGEM‐T‐Easy vector (Promega, Madison, WI, USA). The amplification primers used were: *pri‐mir‐7‐1* A175T‐1st PCR, 5′‐AAA ACT GCT GCC AAA ACC AC‐3′ and 5′‐ACA GCA CCA ATC ATT TGA CCT GTA GAG GCA TGG CC‐3′; *pri‐mir‐7‐1* A175T‐2nd PCR, 5′‐AAA ACT GCT GCC AAA ACC AC‐3′ and 5′‐GCT GCA TTT TAC AGC ACC AA‐3′. These plasmids were linearized with *Spe*I, *Pst*I or *Sal*I and used for *in vitro* transcription by T7 RNA polymerase (M0251S; New England Biolabs, Ipswich, MA, USA). After plasmid digestion by DNaseI (Promega), pri‐miRNA probes were used for m^6^A modification *in vitro*. pri‐miRNA probes (200 ng) were mixed with 200 pmol of S‐adenosylmethionine (B9003S; New England Biolabs) and 500 ng of BSA (B9000; New England Biolabs) in reaction buffer [20 mm Tris‐HCl, pH 7.5, 0.01% Triton X‐100, 1 U·μL^−1^ recombinant RNase inhibitor (2313A; TaKaRa, Kusatsu, Japan) and 1 mm dithiothreitol]. Subsequently, 25 ng of recombinant NF90 and NF45 were added to the reaction mix and incubated at 4 °C for 15, 30 and 60 min, followed by the addition of 50 ng of recombinant human METTL3 + METTL14 protein (Active) (ab268789; Abcam, Cambridge, UK) (total volume of 10 μL) and incubation at 37 °C for 60 min to modify pri‐miRNA m^6^A levels. These reaction mixes were denatured by heating at 95 °C for 3 min, followed by chilling on ice. The m^6^A modified pri‐miRNA probes were spotted on an Amersham Hybond‐N+ membrane (RPM303B; Cytiva, Marlborough, MA, USA).

Subsequently, the pri‐miRNA probes were UV cross‐linked in a UV‐transilluminator (UVP; Analytik Jena GmbH, Jena, Germany). The membrane was washed with 1× PBS buffer containing 0.1% Tween 20 (PBST), blocked with 5% skim milk in PBST and incubated with anti‐m^6^A antibody (dilution 1 : 500; Product No. 202‐003; Synaptic Systems, Goettingen, Germany) overnight at 4 °C. After incubating with horseradish peroxidase‐conjugated anti‐rabbit IgG secondary antibody (Product No. 7074; Cell Signaling Technology, Danvers, MA, USA), the membrane was visualized using ImmunoStar LD (296‐69901; Wako).

The membrane was stained with 0.1% methylene blue solution (19‐3240‐6; Merck) to ensure that an equal number of pri‐miRNA probes were spotted on the membrane. Images were captured and the intensities of specific bands were measured using a FUSION chemiluminescence imaging system (Vilber Bio Imaging).

### Quantification of m^6^A RNAs using an *in vitro* m^6^A modification assay

*pri‐mir‐7‐1* probes (200 ng) were mixed with 200 pmol of *S*‐adenosylmethionine and 500 ng of BSA in the reaction buffer. Subsequently, 25 ng of recombinant NF90, NF45 and/or 50 ng of recombinant human METTL3 + METTL14 protein (Active) were added to the reaction mix (total volume of 10 μL), incubated at 37 °C for 60 min, denatured by heating at 95 °C for 3 min and chilled on ice. The m^6^A quantification of pri‐miRNA probes was conducted using a colorimetric m^6^A RNA methylation assay kit (ab185912; Abcam) in accordance with the manufacturer's protocol.

### Immunoprecipitation

Whole cell extracts from HEK293 cells were prepared using cold lysis buffer (50 mm Tris‐HCl, pH 8.0, 150 mm NaCl, 5 mm EDTA, 0.5% NP‐40, 0.1 mm phenylmethylsulfonyl fluoride, and proteinase inhibitor mixture; 03969‐21; Nacalai Tesque, Kyoto, Japan). The whole cell extracts were immunoprecipitated with anti‐FLAG M2 affinity gel (A2220‐1ML; Sigma‐Aldrich, St Louis, MO, USA). The immunoprecipitates were eluted by the addition of 40 μL of 3 × FLAG peptide (100 μg·mL^−1^; F4799; Sigma‐Aldrich) and analyzed after immunoblotting.

### Bioinformatic analysis

A sequence‐based RNA adenosine methylation site predictor (SRAMP; http://www.cuilab.cn/sramp) [19] was used to predict potential m^6^A modification sites on *pri‐miR‐7‐1*. The sequence of *pri‐miR‐7‐1* is shown in Fig. S1A.

## Results

### NF90‐NF45 impairs the ability of METTL3/14 to facilitate *miR‐7* biogenesis

**Fig. 1:**
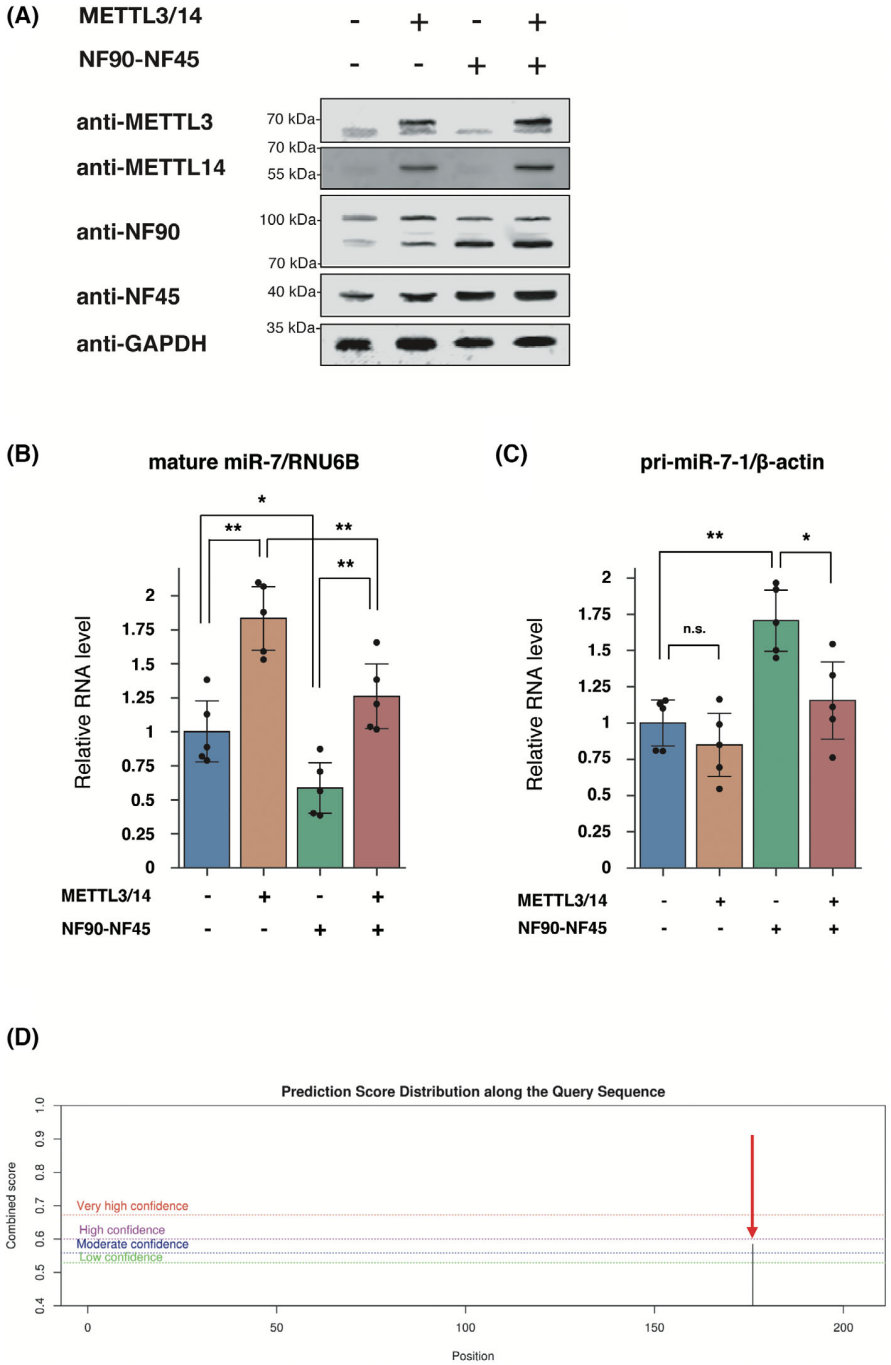
Co‐expression of METTL3/14 overcomes the NF90‐NF45‐induced accumulation of *pri‐mir‐7‐1*. (A) METTL3/14 and NF90‐NF45 expression levels detected by immunoblotting. GAPDH was used as an internal control. Experiments were performed independently in triplicate, and representative results are shown. (B and C) Mature *miR‐7* (B) and *pri‐mir‐7‐1* (C) levels in cells transfected with METTL3/14 and/or NF90‐NF45 expression plasmids or mock plasmids analyzed by qRT‐PCR. *RNU6B* and *β‐actin* were used as an internal control to normalize the data. Data are presented as a scatter plot (*n* = 5 per group) and expressed as the mean ± SD. *P < 0.05, **P < 0.01 relative to the mock transformant according to a two‐tailed Welch's *t* test. n.s., non‐significant. (D) Potential targeted m^6^A motif sites in *pri‐mir‐7‐1* according to the SRAMP online website. The horizontal and vertical axes represent the nucleotide positions within *pri‐miR‐7‐1* (1–203 nucleotides) and the probability score for m^6^A modification (combined score), respectively. The red arrow indicates an adenosine at position 175 (A175) within *pri‐mir‐7‐1* with a high probability of m^6^A modification.

Immunoblot analysis confirmed high METTL3/14 and NF90‐NF45 expression in HCC cell line HepG2 cells overexpressing these protein complex (Fig. 1A). The expression level of mature *miR‐7* (an anti‐oncogenic miRNA: anti‐oncomiR) was significantly elevated by over 1.5‐fold in METTL3/14‐overexpressing cells compared to that in control cells transfected with a mock plasmid, which is the same plasmid backbone used for overexpression of METTL3/14 without these genes (Fig. 1B, orange bar). By contrast, overexpression of NF90‐NF45 significantly reduced the level of mature miR‐7 (Fig. 1B, green bar), consistent with our previous finding [15]. Notably, the METTL3/14‐induced elevation of mature miR‐7 was suppressed in the presence of NF90‐NF45 (Fig. 1B, red bar). On the other hand, NF90‐NF45 overexpression led to a significant accumulation of *pri‐mir‐7‐1* (Fig. 1C, green bar), suggesting that NF90‐NF45 functions as a negative regulator of *pri‐mir‐7‐1* processing into mature miR‐7. Importantly, co‐expression of METTL3/14 alleviated the NF90‐NF45‐induced accumulation of *pri‐mir‐7‐1* (Fig. 1C, compare the green and red bars), indicating that METTL3/14 may counteract the inhibitory effect of NF90‐NF45 on pri‐miRNA. These results raise the possibility that METTL3/14‐induced m^6^A modification of the *pri‐mir‐7‐1* could overcome the repression of its processing by NF90‐NF45. Indeed, the *pri‐mir‐7‐1* sequence contains a DRACH motif, which is a target site for METTL3/14 (Fig. S1A). A putative m^6^A modification site (adenosine at position 175: A175) within *pri‐mir‐7‐1* was predicted (Fig. 1D, a red arrow) using SRAMP (http://www.cuilab.cn/sramp) [19]. The modification of this site by m^6^A is also registered in the m^6^A modification database RMBase v2.0, which includes m^6^A‐seq data (https://rna.sysu.edu.cn/rmbase) (ModID: m^6^A_site_451212). Furthermore, we carried out *in vitro* m^6^A modification assay probed with *pri‐mir‐7‐1* with or without mutation of A175. Replacement of A175 by thymine (A175T) in *pri‐mir‐7‐1* causes a significantly decrease in m^6^A modification of the pri‐miRNA (Fig. S2A), suggesting that adenosine position 175 of *pri‐mir‐7‐1* has the potential to be methylated by METTL3/14 in cells. Intriguingly, RNAfold analysis (http://rna.tbi.univie.ac.at/cgi‐bin/RNAWebSuite/RNAfold.cgi) showed that the *N* ^6^‐methyladenosine at position 175 of *pri‐mir‐7‐1* evokes large bulge formation harboring single‐stranded RNA (Fig. S3). NF90‐NF45 associates with double‐stranded RNA, whereas METTL3/14 recognizes single‐stranded RNA. Therefore, METTL3/14‐induced m6A modification of A175 on *pri‐mir‐7‐1* may prevent NF90‐NF45 from the binding to *pri‐mir‐7‐1* through the formation of bulged structure. Inversely, the association of NF90‐NF45 with double‐stranded region of non‐methylated *pri‐mir‐7‐1* would disturb the accessibility of METTL3/14 to the *pri‐mir‐7‐1*. Collectively, these findings imply that METTL3/14‐mediated m^6^A modification at position 175 of *pri‐mir‐7‐1* may positively control the biogenesis of *miR‐7*.

To confirm the effect of METTL3/14 and NF90‐NF45 on endogenous *pri‐mir‐7‐1* processing in the HCC cell line, we measured the level of exogenous *pri‐mir‐7‐1* and mature miR‐7 in HEK293 cells co‐overexpressing *pri‐mir‐7‐1*, METTL3/14 and NF90‐NF45.

**Fig. 2:**
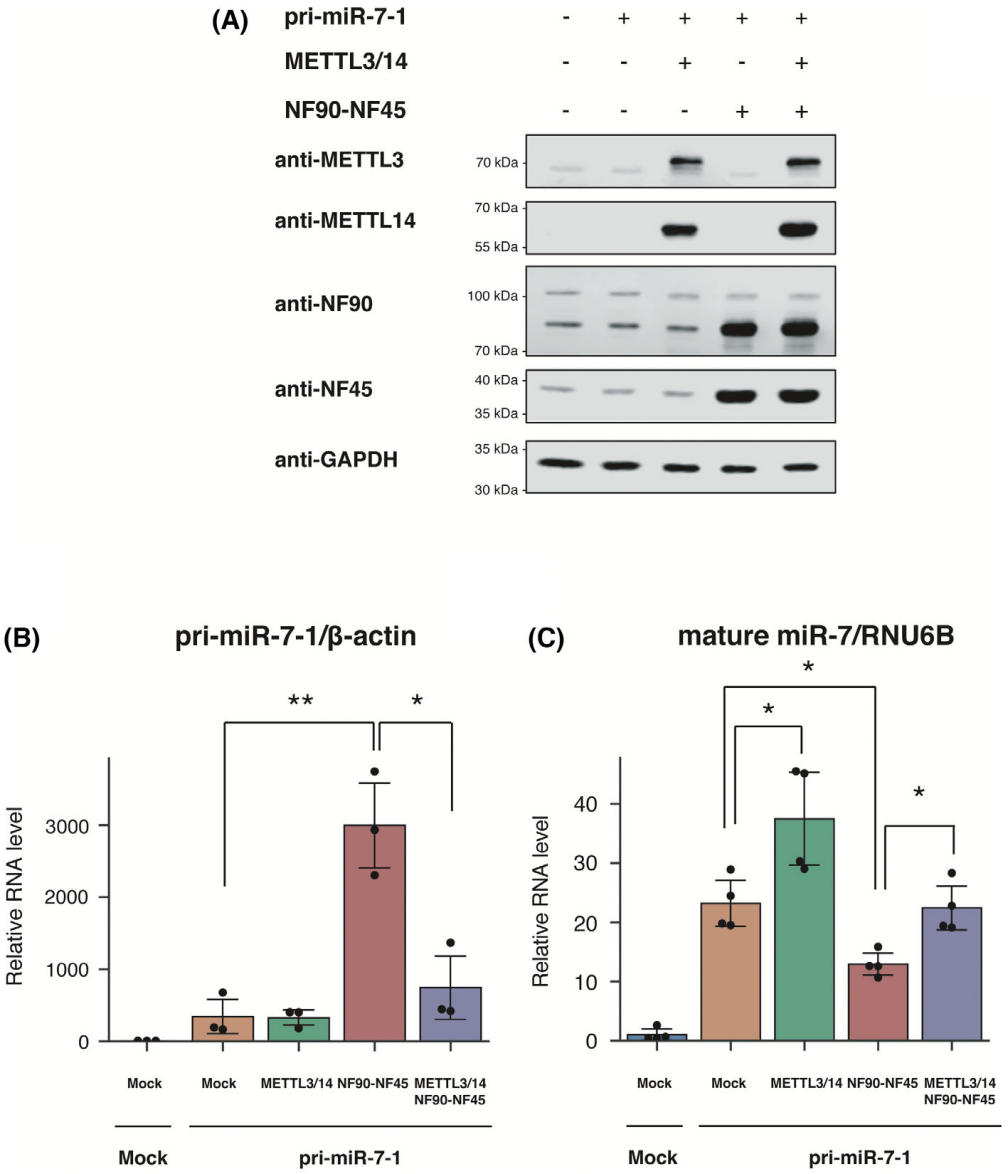
Competition between the NF90‐NF45 and METTL3/14 complexes affects miR‐7 biogenesis. (A) HEK293 cells were transfected with *pri‐mir‐7‐1*, NF90‐NF45 and/or METTL3/14 expression plasmids. The expression levels of NF90, NF45, METTL3 and METTL14 were detected by immunoblotting. GAPDH was used as an internal control. Experiments were performed independently in triplicate, and representative results are shown. (B and C) RNA levels of *pri‐mir‐7‐1* (B) and mature *miR‐7* (C) in cells transfected with the indicated expression plasmids analyzed by qRT‐PCR. *β‐actin* and *RNU6B* were used as internal controls to normalize the data. Data are presented as a scatter plot and expressed as the mean ± SD [*n* = 3 (B) or 4 (C) per group]. *P < 0.05, **P < 0.01 relative to the sample indicated in the Figure, using a two‐tailed Welch's *t* test.

Immunoblotting confirmed the overexpression of METTL3/14 and NF90‐NF45 (Fig. 2A). Furthermore, qRT‐PCR analysis showed that there was no difference in *pri‐mir‐7‐1* expression between mock cells and cells overexpressing METTL3/14, whereas *pri‐mir‐7‐1* accumulated in cells overexpressing NF90‐NF45 (Fig. 2B, red bar). Importantly, co‐overexpression of METTL3/14 and NF90‐NF45 significantly prevented the effect of NF90‐NF45‐induced pri‐miRNA accumulation (Fig. 2B, purple bar). Meanwhile, NF90‐NF45 overexpression led to the suppression of mature *miR‐7* production (Fig. 2C, red bar). Furthermore, METTL3/14 overexpression significantly recovered the NF90‐NF45‐induced inhibition of mature *miR‐7* production (Fig. 2C, purple bar). These results show that METTL3/14 relieves the inhibition of miR‐7 biogenesis by NF90‐NF45.

### NF90‐NF45 inhibits m^6^A modification of *pri‐miR‐7‐1* by METTL3/14 *in vitro*

**Fig. 3:**
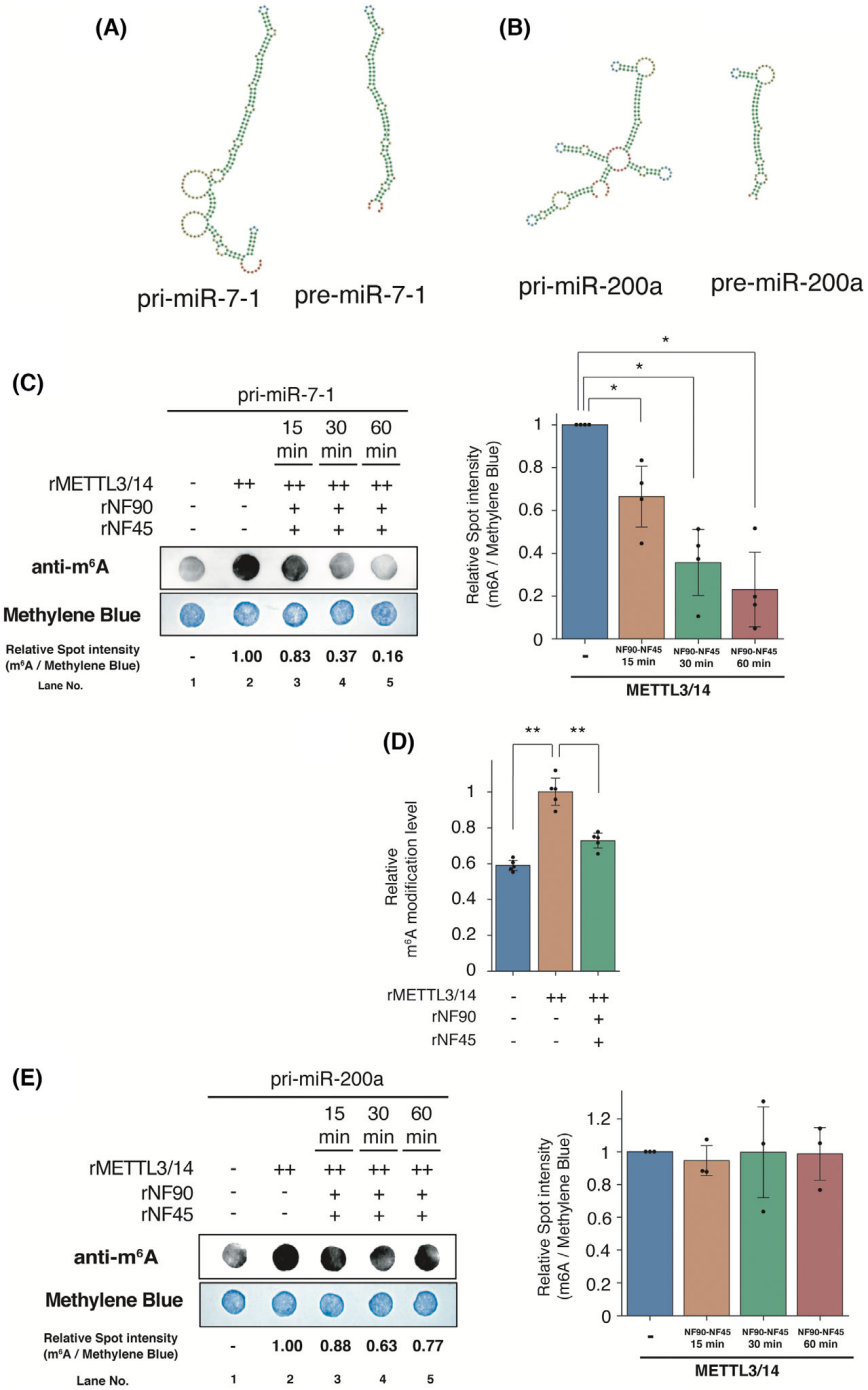
NF90‐NF45 complex impairs m^6^A modification of *pri‐mir‐7‐1* by METTL3/14 owing to preferential binding of NF90 to pri‐miRNAs *in vitro*. (A and B) Predicted structural models of *pri‐mir‐7‐1*/*pre‐mir‐7‐1* (A) and *pri‐mir‐200a*/*pre‐mir‐200a* (B) by RNAfold (http://rna.tbi.univie.ac.at/cgi‐bin/RNAWebSuite/RNAfold.cgi) using the minimum free energy (MFE) model. Temperature conditions and salt concentration were 37 °C (default) and 1.021 m (default), respectively. The models were represented by FORNA (http://rna.tbi.univie.ac.at/forna). (C and E) *In vitro* m^6^A modification assay performed using *pri‐mir‐7‐1* (C) and *pri‐mir‐200a* (E) probes and recombinant METTL3/14, NF90 and NF45 proteins. The levels of m^6^A modification on *pri‐mir‐7‐1* or *pri‐mir‐200a* were detected by immunoblotting and standardized by methylene blue staining. The spot intensities were measured by densitometry and presented as a bar graph. Data are presented as a scatter plot and expressed as the mean ± SD [*n* = 4 (C) or 3 (E) per group]. (D) The level of m^6^A modification in *pri‐mir‐7‐1* probes was analyzed using the m^6^A RNA methylation assay quantification kit in accordance with the manufacturer's protocol (ab185912; Abcam). The assay was performed using a *pri‐mir‐7‐1* probe and recombinant METTL3/14, NF90 and NF45 proteins. Data are presented as a scatter plot and expressed as the mean ± SD (*n* = 5 per group). *P < 0.05, **P < 0.01 relative to control using a two‐tailed Welch's *t* test. ‘+’ and ‘++’ indicate 25 and 50 ng of recombinant protein, respectively.

We previously found that NF90‐NF45 or NF90 alone competes with DGCR8 (a responsible factor for the pri‐miRNA processing in miRNA biogenesis) binding to pri‐miRNA [13, 16]. The data shown in Figs 1 and 2 open the possibility that the binding of NF90‐NF45 to pri‐miRNA may impair the accessibility of METTL3/14 to pri‐miRNA, resulting in reduced m^6^A modification of pri‐miRNA. Because m^6^A modification of pri‐miRNA accelerates pri‐miRNA processing [10, 12], it is assumed that reduced m^6^A modification of pri‐miRNAs leads to the inhibition of miRNA biogenesis. This hypothesis was tested using *in vitro* m^6^A modification assays probed with *pri‐mir‐7‐1*, *pri‐mir‐186* and *pri‐mir‐200a*. *pri‐mir‐200a* has many more bulges compared to that of *pri‐mir‐7‐1* and *pri‐mir‐186* (Fig. 3A,B and Fig. S2E). Moreover, we previously found that NF90 weakly binds to *pri‐mir‐200a* compared to pri‐miRNAs harboring highly stable structures with few bulges [16]. *pri‐mir‐7‐1*, *pri‐mir‐186* and *pri‐mir‐200a* were methylated at the *N* ^6^ position of adenosine *in vitro* (Fig. 3C,E and Fig. S2D, lane 2). Indeed, the DRACH sites that are targeted by METTL3/14 are contained in *pri‐mir‐7*, *pri‐mir‐186* and *pri‐mir‐200a* (Fig. S1A). Notably, m^6^A RNA methylation of *pri‐mir‐7‐1* and *pri‐mir‐186* significantly decreased following the addition of NF90‐NF45 (Fig. 3C and Fig. S2D, lanes 3–5). We also evaluated the quality of *pri‐mir‐7‐1* accompanying the reaction in this assay to ascertain whether the probe is degraded during the reaction. As shown in Fig. S2B, the degradation of *pri‐mir‐7‐1* did not occur during the reaction with or without NF90‐NF45 and METTL3/14, indicating that the reduction in m^6^A modification of *pri‐mir‐7‐1* following the addition of NF90‐NF45 is not a result of the degradation of *pri‐mir‐7‐1* itself. To confirm the finding shown in Fig. 3C, we also performed a quantification of m^6^A modifications using ELISA. The m^6^A modification of *pri‐mir‐7‐1* was significantly reduced following the addition of NF90‐NF45 and METTL3/14 compared to the addition of METTL3/14 alone (Fig. 3D, compare the orange and green bars). Meanwhile, there was no difference in the m^6^A methylation of *pri‐mir‐200a*, despite the presence of NF90‐NF45 (Fig. 3E, lanes 3–5). Additionally, to confirm the specificity of NF90‐NF45 on impairment in m^6^A modification of *pri‐mir‐7‐1*, we performed an *in vitro* m^6^A modification assay probed with *pri‐mir‐7‐1* using ribosomal protein S3a, which has the ability to be bonded to RNA including 18S rRNA and U11 small nuclear RNA [20], as a control. As a result, recombinant GST‐S3a had no effect on the m^6^A modification of *pri‐mir‐7‐1* (Fig. S2C, compare lanes 2 and 3), whereas the modification was significantly reduced by the addition of NF90‐NF45 (Fig. S2C, compare lanes 2 to 4), suggesting that the NF90‐NF45‐mediated inhibition of m^6^A modification on *pri‐mir‐7‐1* is a specific event. On the other hand, immunoprecipitation analysis showed that there was no interaction between NF90‐NF45 and METTL3/14 (Fig. S1B). These results support the idea that the binding of pri‐miRNAs to NF90‐NF45 impairs the accessibility of METTL3/14 resulting in the inhibition of pri‐miRNA methylation. In conclusion, the inhibition of pri‐miRNA methylation would be partially a result of the binding of NF90‐NF45 to pri‐miRNAs.

## Discussion

The METTL3/14 complex is an RNA methyltransferase that catalyzes m^6^A modification of mRNA and lncRNA in mammals. m^6^A‐modified RNAs are recognized by m^6^A reader proteins, and contribute to RNA decay and stabilization, miRNA biogenesis and regulation of gene expression [2]. Thus, METTL3/14 may contribute to the regulation of multiple physiological processes. Indeed, mice deficient in METTL3 [21] or METTL14 [22] display embryonic lethality, suggesting that these proteins are essential for biological processes. m^6^A modification by METTL3/14 is positively controlled by several associated proteins in mammalian cells: WTAP, VIRMA (i.e. vir‐like m^6^A methyltransferase‐associated protein) (also known as KIAA1429), CBLL1 (i.e. Cbl‐proto‐oncogene‐like‐1), ZC3H13 (i.e. zinc finger CCCH domain‐containing protein 13) and RBM15/15B (i.e. RNA binding motif protein 15/15 paralog) [23]. However, the negative regulator of m^6^A modification by METTL3/14 remains unclear. We previously showed that the overexpression of NF90‐NF45 leads to elevated *pri‐mir‐7* production, which is a primary transcript of anti‐oncogenic miRNAs [15]. NF90‐NF45 exhibits high binding affinity to *pri‐mir‐7* [15]. In the present study, NF90‐NF45 suppressed m^6^A modification of *pri‐mir‐7‐1* by METTL3/14 *in vitro* (Fig. 3C,D). Meanwhile, the m^6^A level of *pri‐mir‐200a* (which exhibits lower binding affinity to NF90 compared to pri‐miRNAs that are highly associated with NF90) was unaltered following the addition of NF90‐NF45 (Fig. 3E). Furthermore, NF90‐NF45 did not interact with METTL3/14 (Fig. S1B). These findings imply that NF90‐NF45 acts as a negative regulator of METTL3/14‐based m^6^A modification of pri‐miRNAs highly associated with NF90. Furthermore, the level of m^6^A pre‐modified *pri‐mir‐7‐1* was not diminished following the addition of NF90‐NF45 *in vitro* (Fig. S1C). This indicates that NF90‐NF45 does not act as an eraser enzyme of m^6^A modification of *pri‐mir‐7‐1*; instead, it competes with METTL3/14 to prevent m^6^A modification on *pri‐mir‐7‐1*.

**Fig. 4:**
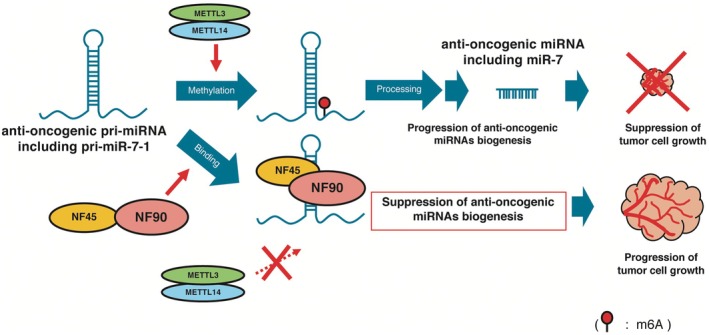
Proposed model for the regulatory mechanism of pri‐miRNA m^6^A modification by NF90‐NF45.

*MiR‐7* exhibits tumor suppressive effects in various types of cancer [24]. Normally, miR‐7 restrains the expression of factors involved in tumorigenesis, including proliferation, cell migration and invasion. Meanwhile, *miR‐7* down‐regulation leads to elevated levels of oncogenic factors, resulting in the promotion of tumorigenesis. We previously found that NF90‐NF45 suppresses the level of *miR‐7* through the inhibition of *pri‐mir‐7‐1* processing in *miR‐7* biogenesis in HCC [15]. The binding of NF90‐NF45 to *pri‐mir‐7‐1* impairs the accessibility of the microprocessor complex to the *pri‐mir‐7‐1*, resulting in inhibition of the processing activity of *pri‐mir‐7‐1* to *pre‐mir‐7* [15]. Similarly, the binding of NF90‐NF45 to anti‐oncogenic pri‐miRNAs including *pri‐mir‐7‐1* would disturb the accessibility of METTL3/14 to the pri‐miRNA, resulting in suppression of anti‐oncogenic miRNAs biogenesis (Fig. 4). Therefore, it is assumed that NF90‐NF45 acts as an oncogenic factor through the impairment of anti‐oncogenic miRNAs in tissues, including *miR‐7* (Fig. 4). Intriguingly, METTL14 enhances m^6^A modification of anti‐oncogenic miRNAs in HCC: *pri‐mir‐126* and *pri‐let‐7* [25]. Furthermore, m^6^A‐modifed *pri‐mir‐126* is recognized by DGCR8, interacts with METTL14 and is processed to mature miRNA, resulting in tumor suppression in HCC [25]. We found that METTL3/14 overexpression facilitates m^6^A modification of *pri‐mir‐7‐1 in vitro* (Fig. 3C,D) and elevates the level of mature *miR‐7* in HCC cell lines (Fig. 1B, orange bar). This suggests that m^6^A‐modified *pri‐mir‐7‐1* may be actively processed to mature *miR‐7*, leading to anti‐oncogenic effects. Taken together, altered expression between NF90‐NF45 and METTL3/14 would play a crucial role in tumorigenesis caused by the down‐regulation of anti‐oncogenic miRNAs (Fig. 4).

NF90 and NF45 bind to many mRNAs, resulting in altered stability and/or translational activity of these mRNAs [26]. The NF90‐NF45 complex binds to oncogenic mRNAs such as *CDK1*, *NUF2*, *CyclinA2* and *Survivin* to enhance their stability [27]. Interestingly, elevated m^6^A levels in oncogenic mRNAs, including *CDK1* [28] and *NUF2*, lead to the decay of these mRNAs in colorectal cancer and renal cell carcinoma [29]. Additionally, NF90 expression is elevated in colorectal cancer [30] and renal cell carcinoma [31]. NF90‐NF45 expression is elevated in various cancers, including non‐small cell lung cancer [32, 33], gastric cancer [34, 35], esophageal squamous cell carcinoma [36] and HCC [15]. These observations suggest that NF90‐NF45 might regulate oncogenic mRNA stability by controlling m^6^A modification via competition with the METTL3/14, leading to tumorigenesis. It is of interest to validate this working hypothesis to help uncover the regulatory mechanism of m^6^A modification by NF90‐NF45 in tumors.

## Conflicts of interest

The authors declare that they have no conflicts of interest.

## Author contributions

TH: conceptualization, methodology, validation, formal analysis, investigation, data curation, writing – original draft preparation, funding acquisition. SM: investigation, validation. KMo: investigation, validation. KMa: writing – review and editing. SA: writing – review and editing. TS: resources, writing – review and editing. SS: conceptualization, project administration, supervision, writing – review and editing.

## Supporting information


**Fig. S1.** NF90‐NF45 does not associate with METTL3/14 and does not function as an eraser of m^6^A modification on pri‐miRNAs.

**Fig. S2.** The NF90‐NF45‐mediated inhibition of m^6^A modification at specific adenosines in pri‐miRNAs is a specific event.

**Fig. S3.** Predicted structural models of pri‐mir‐7‐1 (WT) and A175 m^6^A‐modified pri‐mir‐7‐1 (A175‐m^6^A).

## Data Availability

The data that support the findings of this study are available from the corresponding author upon reasonable request.
