# Supplementary figures and images for: Domain associated with zinc fingers‐containing NF90‐NF45 complex inhibits m6A modification of primary microRNA by suppressing METTL3/14 activity
Source: FEBS Open Bio. 2025 Dec 6;16(5):921–31. doi: 10.1002/2211-5463.70173 (PMC13145339; doi:10.1002/2211-5463.70173)

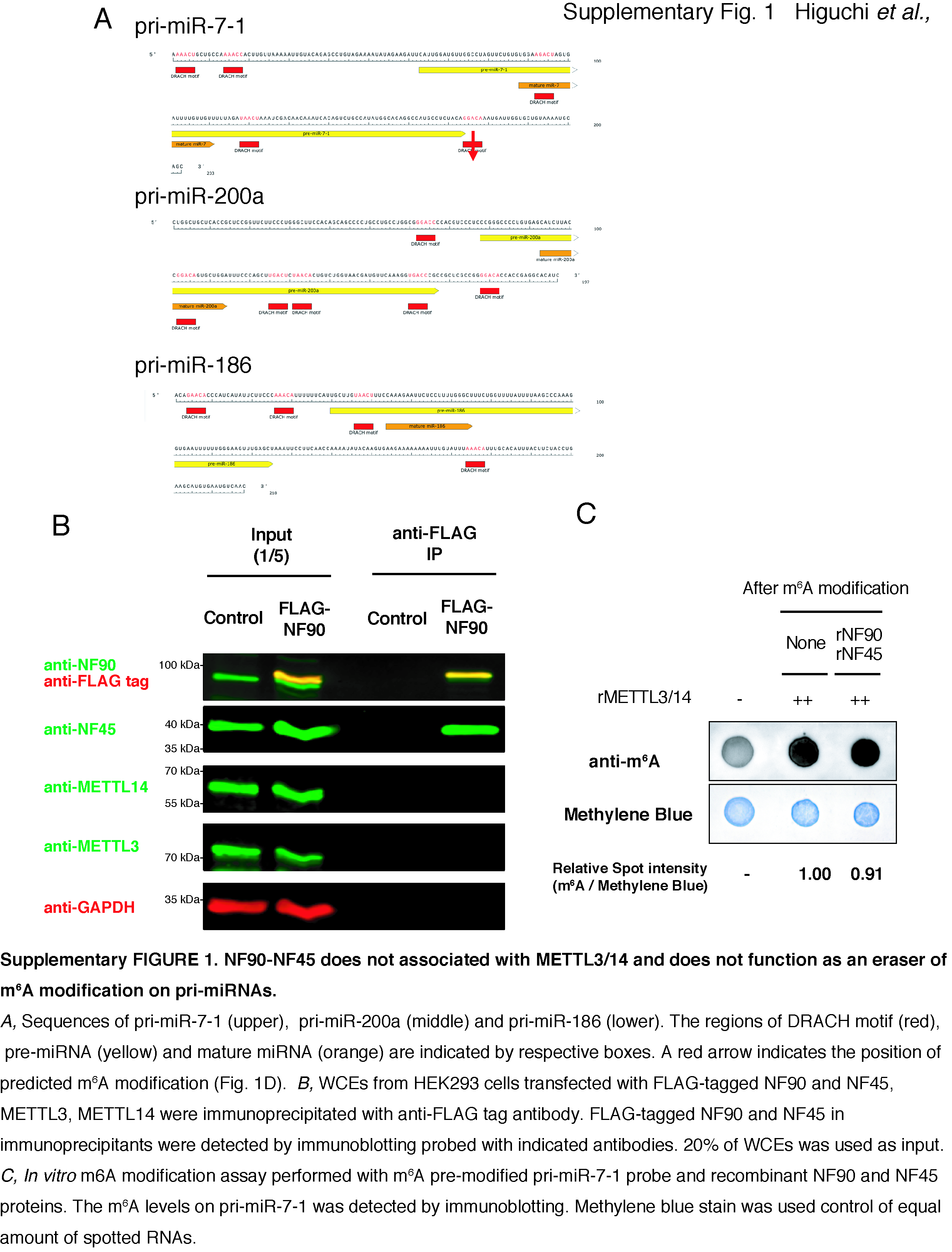

Supplement: Supplementary file 1 — Fig. S1. NF90‐NF45 does not associate with METTL3/14 and does not function as an eraser of m6A modification on pri‐miRNAs. [file FEB4-16-921-s001.tif]

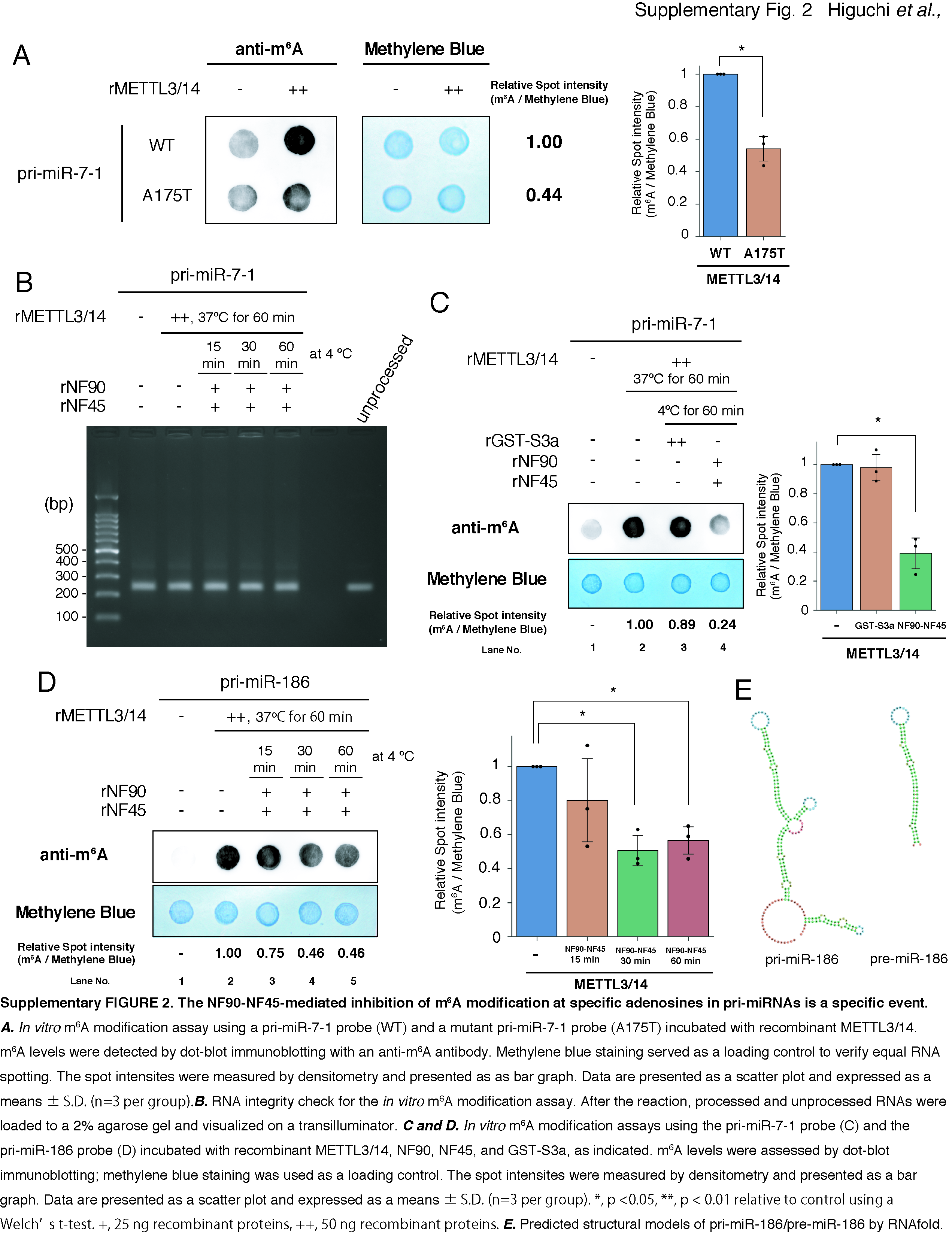

Supplement: Supplementary file 2 — Fig. S2. The NF90‐NF45‐mediated inhibition of m6A modification at specific adenosines in pri‐miRNAs is a specific event. [file FEB4-16-921-s002.tif]

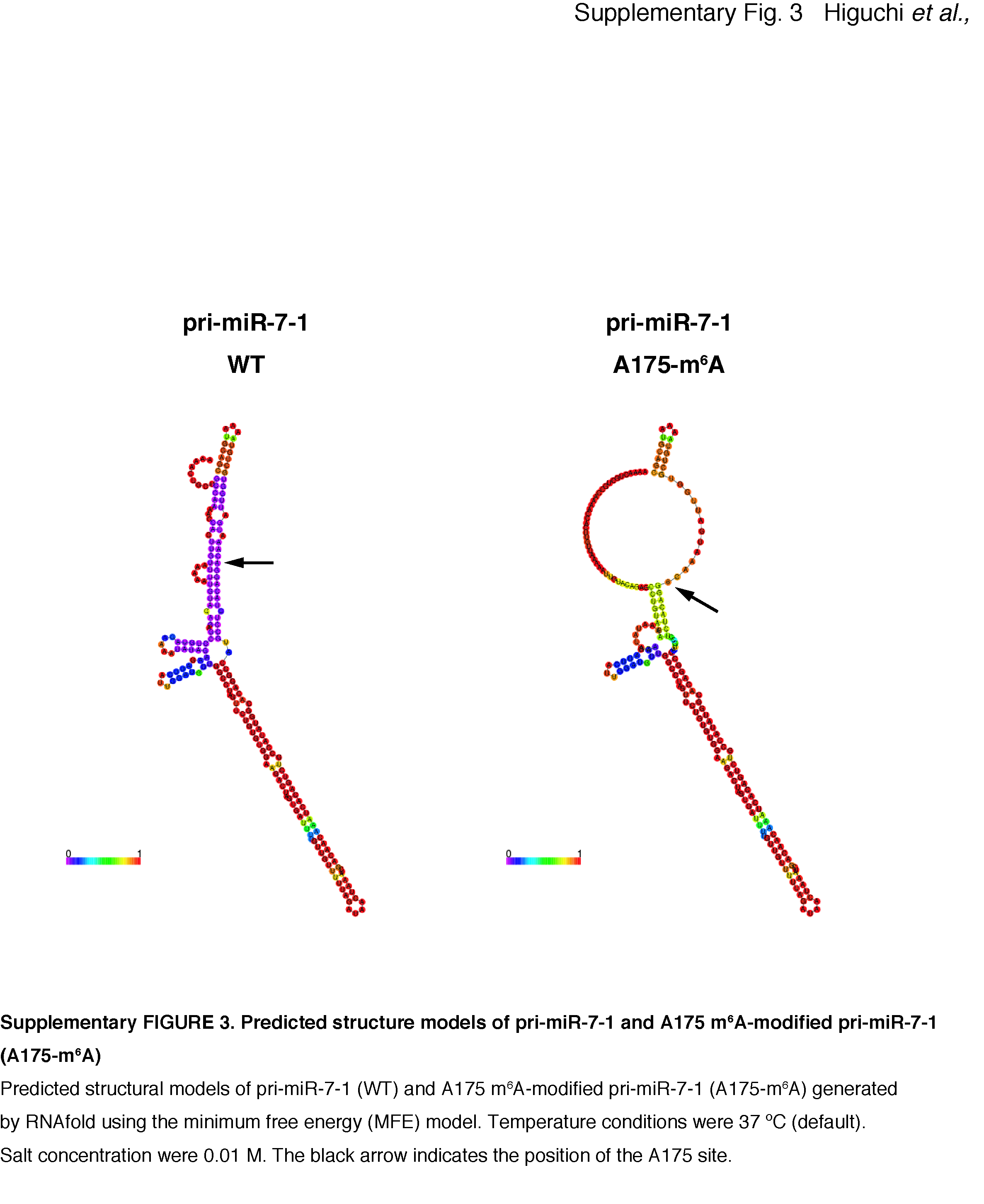

Supplement: Supplementary file 3 — Fig. S3. Predicted structural models of pri‐mir‐7‐1 (WT) and A175 m6A‐modified pri‐mir‐7‐1 (A175‐m6A). [file FEB4-16-921-s003.tif]
